# Prevalence of loneliness and associated factors among older adults at Yilmana Densa District, West Gojjam Zone Amhara region, Ethiopia

**DOI:** 10.3389/fepid.2025.1545342

**Published:** 2025-05-21

**Authors:** Desta Menewab Birhane, Negesu Gizaw Demessie, Abere Woretaw Azagew, Hailemichael Kindie Abate, Chilot Kassa Mekonnen

**Affiliations:** Department of Medical Nursing, School of Nursing, College of Medicine and Health Sciences, University of Gondar, Gondar, Ethiopia

**Keywords:** loneliness, older adults, factors, Yilmana Densa district, Ethiopia

## Abstract

**Background:**

Loneliness is a growing public health issue, particularly among older adults, owing to various internal and external factors related to ageing. However; evidence regarding this segment of the Ethiopian population is scarce. Therefore, this study aimed to assess the prevalence of loneliness and its associated factors among older adults in Ethiopia.

**Methods:**

This community-based cross-sectional study was conducted from April 20 to May 20, 2023. A multistage systematic sampling technique, using an interviewer-administered questionnaire, was used. Data were entered into Epi Data version 4.6.0.0 and exported to the Stata version 14 software for analysis. A binary logistic regression analysis was conducted. Variables with a *p*-value < 0.20 in the Bivariable analysis were entered into multivariable regression and variables with a *p*-value < 0.05, with a 95% confidence interval (CI) were considered statistically significant.

**Results:**

A total of 840 older adults took part with a 99.2% response rate. The overall prevalence of loneliness among older adults was 48.69%, with 95% CI = 45.31–52.07%. Living alone (OR=2.59, 95% CI = 1.11–6.05), the presence of chronic illness (OR = 1.69, 95% CI = 1.12–2.54), sleep time greater than 9 h (OR = 1.56, 95% CI = 1.08–2.22), impairment (OR = 5.09, 95% CI = 3.17–8.19), and poor social support (OR = 4.38, 95% CI = 2.53–7.59) were positively, but family size <5 (OR = 0.62, 95% CI = 0.45–0.85) and good health status (OR = 0.43, 95% CI = 0.27–0.66) were negatively associated with loneliness among older adults.

**Conclusions:**

Nearly half of the participants felt lonely. Hence, every concerned body should pay special attention to this sidelined segment of the population by creating better social support networks, providing a conducive living environment, and providing aid to impaired older adults.

## Introduction

### Background

The global population aged 60 years or over numbered 962 million in 2017 and is expected to double again by 2050, when it is projected to reach nearly 2.1 billion (1). This segment of the population passes through different physical, mental and psycho-social problems due to ageing. Of these problems, loneliness takes a lion's share and is becoming a growing public health issue (1, 2). Its burden is growing, particularly among older adults, owing to various internal and external factors related to ageing (3). It is defined as a subjective feeling which encompasses unpleasantness, sadness, emptiness, distress, suffering, isolation, lack of meaning, unwanted feelings, non-belongingness, and lack of companionship (4, 5). There is a significant mismatch between actual and desired (or ideal) social connections and not merely social isolation. In other words, one might feel lonely despite enjoying a large social network and a high number of social connections (6–8). Social isolation is an objective description of a lack of social connections. Although social isolation may lead to loneliness, these terms are not interchangeable (7, 8). Loneliness is widely recognized as a public health issue with a prevalence ranging from 10.5% to 34% in the elderly global population (9, 10).

Loneliness may develop in individuals of all ages, but it is a crucial issue in older adults and has become a serious public health concern (11, 12). Multiple factors contribute to the increased loneliness among older adults. In fact, due to changes in their life cycle stages, such as retirement or age-related losses, declining health status, and decreased physical activity, older adults experience loneliness and social isolation (13). The prevalence of loneliness in older adults (aged 60 years and older) across European countries has shown: Ukraine (34.0%), Russia (24.4%), Hungary (21.1%), and Poland (20.1%) (14). A study conducted in the United Kingdom showed that over one million older people (aged greater than 60 years) say they always or often feel lonely, and half (49%) of all people aged 75 and over live alone (15). A study in the United States showed that approximately 17%–57% of people feel lonely, especially older adults suffering from anxiety, depression, and dementia (16). A study conducted in South African countries shows that approximately 10% of older adults report being lonely (17).

Evidence shows that loneliness in older adults is a major risk factor for broad-based morbidities, both psychological and physical, including depression, physical health, anxiety, sleep disturbances, unhealthy behaviour (smoking, excessive use of alcohol, and substance abuse), cognitive impairment, and increased obesity and diabetes challenges, as well as the risk of death in later life (13, 14, 16). In addition, chronic illnesses such as Alzheimer's disease, vascular resistance, and high blood pressure, as well as chronic diseases such as metabolic disorders, cardiovascular disorders, hypertension, lung disease, eating disorders, obesity, and premature mortality are associated with loneliness (15, 17, 18).

Several studies show that loneliness is influenced by a wide range of factors, such as socio-demographic, and psychosocial, with gender, age, marital status, living arrangement, education level, family income, health-related behaviours, health status, and so on and having little contact with significant friends or low-quality friendship ties; worsening physical health (e.g., increased chronic illness and impaired mobility); and lacking socioeconomic resources (e.g., limited education and low income) (8, 14, 17). In addition, a study conducted in Ireland showed that loneliness is influenced by individual characteristics of the social environment, such as having no children, reality/place of residence, length of residence, and older age (19).

Despite older adults being highly vulnerable to loneliness, and their global numbers having increased; less attention has been given to them. However, loneliness contributes to multiple morbidities and mortalities related to physical and mental illnesses. Older adults are highly influenced by underlying factors such as unemployment/retirement, age-related diseases, and loss of relatives/friends; as a result, they are prone to loneliness. Despite several studies being conducted in developed countries and a few in African countries, there has been no documented evidence of this problem at the time of this study. The results of this study will help health administrators and policymakers formulate interventions to provide need-based healthcare service plans and budget allocation. They should also help service providers to understand the factors that cause loneliness and provide hints for intervention. This study serves as baseline information for educators, researchers, and stakeholders. Although numerous studies have been conducted among older adults in developed countries and a few in African countries, only a limited number of studies have been conducted in Ethiopia. In addition, it is influenced by sociocultural and socioeconomic factors; therefore, it is necessary to study this problem in this study area. Therefore, this study aimed to assess the prevalence of loneliness and its associated factors among older adults in the Adet District.

## Methods

### Study area, study design, and period

A community-based cross-sectional study was conducted from April 20 to May 20, 2023, in the Yilmana Densa district of the West Gojjam Zone in the Amhara Regional State. It is 42 km from Bahir Dar, the capital city of Amhara National Regional State, and 524 km from Addis Ababa, the capital city of Ethiopia. According to the 2015 population projection in Ethiopia, the total population of the Yilmana Densa district had 40 Kebeles estimated to be 286,458 people. Among these, the study population in the selected 10 kebeles had a total of 5,730 households. The district currently consists of 40 kebeles; it has one primary hospital, 10 health centres, and 40 health posts.

### Source and study population

All older adults aged ≥60 years living in the Yilmmana Denssa district were the source of the population. All older adults ≥60 years of age living in the selected kebeles of the Yilmmana Denssa district were considered as the study population.

### Inclusion and exclusion criteria

All older adults living in the Yilmana Densa district for at least 6 months and having residential identification cards were included in the study. Participants who were not registered as permanent residents and those who did not have residential identification cards were excluded from this study.

### Sample size determination and sampling procedures

The sample size was determined by using a single population proportion formula with the assumption of a 50% proportion, 95% confidence level, and 5% margin of error, and a 10% nonresponse rate was added. Based on this, the actual sample size for this study was 385 and, with the assumption of non-response, 10% was added to generate an initial sample of 424.

$$n=\frac{{\left[\frac{Z\alpha }{2}\right]}^{2}*\;P[1-P]}{D^{2}}$$

*n* = initial sample size

Z*_α_*_/2_ = 1.96, the corresponding Z-score for the 95% CI

P = proportion = 50%

D = margin of error=5% = 0.05

$$n=\frac{{\left[1.96\right]}^{2}\;*\;[.5\;*\;.5\}}{0.05^{2}}=384.16=385$$

As per the steps taken to reach respondents, we used a 2-design effect to obtain a final sample size of 847. The list of study participants was obtained from the district health office using health extension workers' registration books. A simple random technique was used to select ten kebeles; then, the calculated sample size was proportionally allocated to each kebele based on the number of households. Finally, study participants were selected using a systematic random sampling technique at every seventh interval using family folders from health extension workers by the first participants selected using the lottery method (Supplementary Figure S1).

### Study variables

#### Dependent variable

Loneliness was the only dependent variable.

#### Independent variables

##### Socio-demographic factors

These include age, marital status, educational level, occupational status, monthly income, living arrangements, and residency.

##### Behavioural factors

These were alcohol use, regular exercise, smoking, and sleeping time.

##### Health-related factors

The presence of chronic diseases, self-rated health status, mobility, visual impairment, and hearing impairment.

### Operational definition of variables

#### Loneliness

We the UCLA-3 measuring tool to measure loneliness with the ratings (Never = 0) to (4 often) that contained 20 items with four Likert scale response formats (0 = never feel lonely, 1 = rarely feel lonely, 2 = sometimes feel lonely, and 3 = often feel lonely) using the mean of the total score answered by the respondents. This results, in a minimum of 0 (if all answers are “Never”) to a maximum of 80 (if all answers are “Often”). The interpretation of the total score (0–19 = very low loneliness,(20–39 = moderate loneliness), (40–59 = high loneliness), and (60–80 =very high loneliness). Thus, a code of “1” was given for older adults who had loneliness equal to and above the mean (≥24.73) and a code of “0” was given for those older adults who were not classed as having loneliness, with a score below the mean score (<24.73) (20, 21). The mean was used because of data distribution in which the sum was normally distributed and we used the mean over the median. Although, the mean and median will be similar, but making the mean a good representative measure in this case. Moreover, the sum of loneliness was continuous and unbiased data, hence we need to capture the overall magnitude of values rather than just middle value.

#### Alcohol use

This was assessed by asking participants, “During the past seven days, did you drink alcohol?” (Classified as “Yes” or “No”). Those who responded “yes” were asked: “During the past seven days, how many drinks of any alcoholic beverage did you have each day?” Risky alcohol use was classified as ≥10 drinks consumed in the past week (17).

#### Chronic disease

The presence of long-term illness (congestive heart failure, respiratory disease, diabetes, renal disease, HIV/AIDS, HTN, epilepsy) which is assured by a doctor. Self-report of one of these illnesses was noted as “Yes”, while the absence of these conditions was denoted as “No” (13).

#### Social support

This was measured using the Oslo-3 Social Support Scale (OSSS-3) scores ranged from 3 to 14, with a score of 3–8 indicating poor support, 9–11 indicating moderate support, and 2–14 indicating strong support (22).

#### Regular exercise

Individuals who worked out or walked for 30 min a day or at least 3 days a week were considered performers of physical exercise (17).

#### Self-rate health status

This was measured using a 5-point scale on how participants rated their current health: very good, good, moderate, bad, and very bad. The five categories were collapsed into two categories, 0 = “very good”', “good”, or “moderate” and 1 = “bad” or “very bad” (17).

#### Visual and hearing impairments/loss

These were assessed by asking, “Can you see things even with glasses?” and “Can you hear sounds even with a hearing aid?” They were classed as Yes, “with visual and/or hearing impairments”, or No, “with no visual or hearing impairments” **(**23).

#### Smoking of cigarette

Smoking was assessed by asking, “Do you currently smoke cigarettes?” The options were non-smokers and smokers, respectively. Elderly individuals who smoked once a week were considered smokers (24).

### Data collection tool and procedures

A questionnaire pre-test was conducted before the main data collection period to assess its clarity, relevance, and reliability. For this purpose, 5% (42) of the total study population were selected for the pretest. Based on the pretest, the necessary modifications, such as language clarity and length of the questionnaire, were corrected to ensure the questionnaire well understood and contextually appropriate. The final version was also validated and used for data collection in the main study. Data collection was done by using a face-to-face interviewer-administered questionnaire that was adapted from different literature (20, 21). The questionnaire consisted of four parts (socioeconomic and demographic characteristics, health-related factors, and behaviour-related factors) and loneliness-related questions. The outcome variable was measured using the UCLA revised version-3 loneliness measurement tool (20, 21).

The questionnaire was translated into the local language of Amharic by language experts and translated back into English by another person to ensure consistency and clarity. Before data collection, a pre-test was performed to determine the consistency of the information. Data were collected by eight BSc degree nurses, and supervision was performed by two MSc nurses during the control data collection process. Missing data was managed through on-the-spot checking and cleaning throughout the data collection period by the supervisors.

### Data quality control

To ensure the quality of data, a pre-test was done with a 5% sample size (with 43 older adults) in East Gojjam, Motta Town, before the actual data collection. Two-day training was provided to data collectors and supervisors about the purpose of the study, face-to-face interviews, interviewing techniques, and maintaining the privacy and confidentiality of the respondents.

### Data processing and analysis

The questionnaires were checked for completeness and consistency. The data were entered into Epi data 4.6.0 versions and then exported to Stata version 14 for analysis. Descriptive and summary statistics are presented in the form of text, tables, and graphs. Bivariable logistic regression analysis was performed to select candidate variables for multivariable analysis. Then those, variables with a *P*-value < 0.20 were taken as candidates for multivariable analysis. Finally, multivariable logistic regression analysis was performed to control for the possible confounding effect of the selected variables, and variables with a *P*-value < 0.05, taken as statistically significant association with loneliness; OR with a 95% confidence interval (CI) used to show the degree of association between the independent and outcome variables. Multicollinearity between independent variables was assessed using the variance inflation factor (VIF). The Variance Inflation Factor (VIF) was used to assess multicollinearity in the regression model, indicating how much a coefficient's variance is inflated due to correlation with other predictors. VIF values range from 1 (no multicollinearity) to 10 (serious multicollinearity). In this study the VIF ranged from 1.01 to 4.87, the highest was observed in variables of age and having a chronic illness with VIF of 4.64 and 4.87 respectively. But, the mean VIF was 2.907 which indicate there was no severe multicollinearity. For a finally fitted multivariable logistic regression model, model fitness was checked using the Hosmer-Lemeshow goodness-of-fit with a *P*-value of 0.83, which showed the model fitted to the outcome variable.

## Results

### Socio-demographic characteristics

A total of 840 older adults participated in this study with a response rate of 99%. Nearly half (49.17%) of the respondents were within the age range of 65–69 years. More than half (59.64%) of the respondents were male. Two-thirds (67.02%) of respondents were married. In addition, the most of respondents (86.07%) were orthodox followers. Nearly half, of the respondents (47.98% and 48.93%) couldn't read or write and were farmers, respectively. The majority (82.86%) of respondents lived with spouses. More than two-thirds (69.76%) of respondents had five or less than five family members. Moreover, one-fourth (25.71%) of respondents had poor social support (Table 1).

**Table 1:** Socio-demographic characteristics of older adults at Yilmana Densa Adet District, West Gojjam, Ethiopia, 2023 (*n* = 840).

Variables	Categories	Frequency	Percentage
Age in years	60–64	23	2.74
	65–69	413	49.17
	70–74	216	25.70
	75–79	145	17.26
	≥80	43	5.12
Sex	Male	501	59.64
	Female	339	40.36
Marital status	Married	563	67.02
	Widowed	149	17.74
	Divorced	118	14.05
	Single	10	1.19
Religion	Orthodox	723	86.07
	Muslim	94	11.19
	Protestant	23	2.74
Education level	Can't read and write	403	47.98
	Can read and write	141	16.79
	Primary school	133	15.83
	Secondary school	92	10.95
	College and above	71	8.45
Occupation	Farmer	411	48.93
	Housewife	305	36.31
	Merchant	41	4.88
	Retired	83	9.88
Residency	Rural	394	46.90
	Urban	446	53.10
Living condition	Alone	48	5.71
	With spouse	696	82.86
	With child/children	96	11.43
Family size	<5	586	69.76
	≥5	254	30.24
Monthly income in ETB	<15,000	108	12.86
	15,000–50,000	619	73.69
	≥50,000	113	13.45
Social support	Poor support	216	25.71
	moderate support	361	42.98
	Strong support	263	31.31

### Behavior-related factors

The majority (818; 97.38%) of respondents were not cigarette smokers. Among smokers, more than half (12; 54.55%) smoked three or more than three packs of cigarettes per day. In addition, more than half of the older adults (499; 59.40%) drank alcohol, and of those, more than one-third (39.07%) were drinking 10 or more alcoholic drinks a week. Regarding sleeping time, more than half (503; 59.88%) slept less than 8 h per night (Table 2).

**Table 2:** Behavioral-related factors among older adults at Yilmana Densa Adet District, West Gojjam, Ethiopia, 2023 (*n* = 840).

Variables	Categories	Frequency	Percent (%)
Smoke cigarettes	Yes	22	2.62
	No	818	97.38
Strips per day	<3	10	45.45
	≥3	12	54.55
Drink alcohol	Yes	499	59.40
	No	341	40.60
Amount per week	<10	304	60.92
	≥10	95	39.07
Exercise	Yes	243	28.93
	No	597	71.07
Sleeping time	<7 h	64	7.62
	7–9 h	439	52.26
	≥9 h	337	40.12

### Health-related factors

Less than one-third of older adults (32.26%) had chronic illnesses. Among these, one-third of the older adults had two or more chronic illnesses. In addition, one-third of older adults (32.38%) had a poor self-rated health status, and 8.21% had physical mobility difficulty. Moreover, one-fifth (20.60%) of older adults had hearing and/or visual impairments (Table 3).

**Table 3:** Health behavioral-related factors among older adults at Yilmana Densa Adet District, West Gojjam, Ethiopia, 2023 (*n* = 840).

Variables	Categories	Frequency	Percent (%)
Chronic illness	Yes	272	32.26
	No	568	67.74
Number of chronic illnesses	1	191	70.22
	≥2	81	29.78
Self-rate Health status	Good	575	68.45
	Poor	265	31.55
Impairment of hearing/vision	Yes	173	20.60
	No	667	79.40

### Prevalence of loneliness

Among respondents, 409 (48.69%) of older adults were feeling lonely, with a 95% CI of 45.31–52.07). The mean loneliness score was 24.72, with a standard deviation (SD) of ±13.00. Half of the respondents (431; 51.31%) scored below the mean score.

### Factors associated with loneliness

Sex, occupation, living conditions, education, residency, family size, monthly income, self-rated health status, chronic illness, sleeping time, impairment, and social support were entered into multivariate logistic regression analysis. The analysis showed that living conditions, family size, self-rated health status, chronic illness, sleeping time, impairment, and social support were significantly associated with loneliness.

In this regard, older adults who lived alone had 2.59 times higher odds of feeling lonely compared to those who lived with someone (OR = 2.59, 95% CI = 1.11–6.05). Respondents with five or more family members had 38% lower odds of experiencing loneliness compared to those with fewer than five family members (OR = 0.62, 95% CI = 0.45–0.85).

Respondents with poor social support had 4.38 times higher odds of feeling lonely compared to those with strong social support (OR = 4.38, 95% CI = 2.53–7.59). Besides, older adults with chronic illness had 1.69 times higher odds of experiencing loneliness compared to those without chronic illness (OR = 1.69, 95% CI = 1.12–2.54). In addition, older adults who self-rated their health status as good were 57% times (OR = 0.43, 95% CI = 0.27–0.66) fewer odds of being lonely as compared with those who had poor self-rated health status. Respondents who slept more than 9 h per night had 1.56 times higher odds of experiencing loneliness compared to those who slept 7–9 h (OR = 1.56, 95% CI = 1.08–2.22). Moreover, older adults with hearing and/or vision impairment had 5.09 times higher odds of experiencing loneliness compared to their counterparts (AOR = 5.09, 95% CI = 3.17–8.19). (Table 4).

**Table 4:** Bivariable and multivariable regression analysis of factors for loneliness among older adults at Yilmana Densa Adet District, West Gojjam, Ethiopia, 2023 (*n* = 840).

Variable	Loneliness		With 95% CI COR	AOR
	No loneliness	Loneliness		
Sex				
Female	140 (32.48)	199 (48.66)	1	1
Male	291 (67.52)	210 (51.34)	0.51 (0.38–0.67)	0.83 (0.57–1.20)
Occupation				
Farmer	220 (51.04)	191 (46.70)	1	1
Housewife	144 (33.41)	161 (39.36)	1.28 (0.95–1.73)	1.24 (0.85–1.81)
Merchant	24 (5.57)	17 (4.16)	0.82 (0.42–1.56)	1.24 (0.56–2.76)
Retired	43 (9.98)	40 (9.97)	1.07 (0.66–1.72)	0.95 (0.52–1.75)
Education				
Unable to read & write	212 (49.19)	191 (46.70)	1	1
Able to read & write	68 (15.78)	73 (17.85)	1.19 (0.81–1.74)	1.18 (0.55–1.91)
Primary school	74 (17.17)	59 (14.43)	0.88 (0.59–1.31)	0.96 (0.58–1.56)
Secondary	49 (11.37)	43 (10.51)	0.97 (0.62–1.53)	0.88 (0.50–1.57)
College & above	28 (6.50)	43 (10.51)	1.70 (1.01–2.85)	1.44 (0.76–2.74)
Residency				
Rural	156 (36.19)	238 (58.19)	1	1
Urban	275 (63.80)	171 (41.81)	0.40 (0.30–0.53)	0.71 (0.48–1.06)
Living condition				
With child/children	27 (6.26)	21 (5.13)	1	1
Alone	37 (8.58)	59 (14.43)	**2.05** (**1.02–4.14)**	**2.59** (**1.11–6.05)**
Spouse	367 (85.15)	329 (80.44)	1.15 (0.65–2.07)	1.38 (0.68–2.82)
Family size				
<5	275 (63.81)	311 (75.04)	1	1
≥5	156 (36.19)	98 (23.96)	**0.52** (**0.41–0.75)**	**0.62** (**0.45–0.85)**
Monthly income				
<15,000	37 (8.58)	71 (17.36)	1	1
15,000–50,000	339 (78.65)	280 (68.46)	0.43 (0.28–0.66)	0.83 (0.47–1.47)
≥50,000	55 (12.76)	58 (14.18)	0.54 (0.32–0.95)	1.80 (0.90–3.36)
Social support				
Strong	175 (40.60)	88 (21.52)	1	1
Moderate	218 (50.58)	143 (34.96)	1.30 (0.94–1.84)	0.72 (0.43–1.13)
Poor	38 (8.82)	178 (43.52)	**9.32** (**6.04–14.37)**	**4.38** (**2.53–7.59)**
Alcohol				
No	210 (48.72)	131 (32.03)	1	1
Yes	221 (51.28)	278 (67.97)	2.01 (1.52–2.67)	1.02 (0.68–1.55)
Chronic illness				
No	325 (75.41)	243 (59.41)	1	1
Yes	106 (24.59)	166 (40.59)	**2.09** (**1.55–2.81)**	**1.69** (**1.12–2.54)**
Self-rated Health				
Status				
Poor	243 (56.38)	332 (81.17)	**1**	**1**
Good	188 (43.62)	77 (18.83)	**0.29** (**0.22–0.41)**	**0.43** (**0.27–0.66)**
Sleeping				
7–9 h	251 (58.24)	188 (45.97)	1	1
>9 h	134 (31.09)	203 (49.63)	**2.02** (**1.52–2.69)**	**1.56** (**1.08–2.22)**
<7 h	46 (10.67)	18 (4.40)	0.52 (0.29–0.93)	0.25 (0.18–0.58)
Impairment				
No	395 (91.65)	272 (66.50)	**1**	1
Yes	36 (8.35)	137 (33.5)	**5.52** (**3.71–8.22)**	**5.09** (**3.17–8.19)**

Those bold results are statistically significant findings.

AOR, adjusted odd ratio; COR, crude odd ratio.

## Discussion

The study revealed the prevalence of loneliness among older adults in Yilmana Densa district was 48.69% with 95% CI = 45.31–52.07%. This finding is consistent with studies done in India (48%) (25), Iran (51.77%) (13), five European countries (46%) (26), and 11 European countries (50.50%) (27). The reasons might be the similar study design, study population (age >60 years), and the use of similar measurement tools. However, this finding is higher than studies conducted in the USA (range between 25% and 29%) (14), 43.4% in Germany (28), Taiwan (10.5%) (23), Ireland (33.7%), 17.7% in Australia (29), and South Africa (9.9%) (17). A possible reason for this discrepancy might be due to the different study designs; the studies in Ireland and Taiwan were longitudinal. In addition, it might be due to different measurement tools; a study in India used a single-item question to assess loneliness due to different study periods and cultural variations. In another way, this finding is lower than studies done in Indonesia (64%) (30), China (58.1%) (6), and China (74%) (7). This may be due to variations in the sample size, study setting, measurement tools, study period, and socioeconomic and cultural variations in the study population.

The findings of this study showed that older adults who lived alone had higher odds of experiencing loneliness than those who lived with their children. This finding is consistent with studies done in Indonesia, China, India, and Ireland (6, 19, 25, 30). A reason might be that people who have no relationship with others or live alone feel lonely, and it might be that people who live alone are socially isolated and emotionally lonely. However, this finding contrasts with those of previous studies conducted in Taiwan, China (31), and Korea (32). This finding implies that people living alone do not experience loneliness. This might be due to sociocultural variation. In Ethiopia, living alone is valued highly but leads to loneliness. Therefore, cultural value is a possible reason for the differences in the magnitude of loneliness.

Respondents with five or more family members had 62% less odds of experiencing loneliness as compared with those with less than five family members. In other words, older adults with fewer children experienced loneliness than those with more children. This finding is consistent with those of studies conducted in Indonesia (30), China (9) and Turkey (33). One possible explanation is that older adults with more children experienced greater happiness and life satisfaction, as our community culture values having many children as a sign of wealth and provides more opportunities for receiving affection. Consequently, those with fewer children may suffer from loneliness. Respondents with poor social support had higher of feeling were lonely than those with strong social support. This result is consistent with studies conducted in India (25), Ireland, and China (7, 9), possibly because social support is fundamental for people. As a result, people with poor social relationships develop social isolation which leads to loneliness.

Older adults with chronic illnesses had higher odds of experiencing loneliness as compared to their counterparts. This finding is consistent with studies conducted in Ireland (19), India (25), China (9) and the USA (34). A possible reason might be that people with chronic illnesses are physically incapable of moving, physically compromised, and mostly dependent on others. As a result, they experience loneliness compared to those with no chronic illness.

In addition, older adults who self-rated their health status were 57 times less odds to feel lonely as compared with those with poor health status. This finding is consistent with previous studies conducted in South Africa (17), India (25), China (6) and Ireland (19). A possible explanation might be that older people subjectively rate poor health status as fear, stress, and low self-esteem, as a result of anxiety and social isolation, leading to loneliness.

Older adults who sleep greater than 9 h a night had higher odds of experiencing loneliness than those who sleep for 7–9 h. This finding is consistent with studies conducted in Iran (13) and Ireland (19). This implies the opposite of the line above, which states that those who sleep less are less depressed. Moreover, older adults with hearing and/or vision impairments had higher odds of experiencing loneliness as compared to their counterparts. This finding is consistent with studies done in the USA and South Africa (23, 35). A possible reason might be that hearing and visually impaired older adults require special attention for social connection, and their inability to respond to sound and visual stimuli makes them emotionally lonely. Therefore, district health managers can create better social connections and strong social ties for older adults. Furthermore, special attention must be given to the segments of the population with hearing and visual impairments, those living alone, and those who have no children.

### Strengths and limitations of the study

One of the key strengths of this study is that it is the first of its kind to assess loneliness among older adults in Ethiopia. This pioneering effort provides valuable insights into an often-overlooked public health issue. Additionally, the study employs a statistically large sample size, enhancing the reliability and generalizability of the findings. By shedding light on the prevalence and magnitude of loneliness among older adults, this research contributes to raising awareness about a critical but under-recognized social and psychological concern. As a result, the findings have significant implications for policymakers and stakeholders, offering evidence-based guidance for interventions aimed at improving the well-being of older adults.

However, the study also has certain limitations that should be considered when interpreting the findings. One notable limitation lies in the measurement of loneliness, which was assessed using a mean score derived from participants' responses. While this approach offers a simplified and quantifiable method for analysis, it may not accurately capture the full complexity and variability of individual experiences of loneliness. Specifically, using a mean score can mask important fluctuations or extremes in loneliness levels, potentially leading to either an overestimation or underestimation of the true prevalence and intensity of loneliness among older adults. This methodological choice may therefore limit the depth of insight into the nuanced emotional and social realities faced by the study population. Therefore, we recommended future researchers to go deep into the issue using different measures to figure out the severity and true prevalence of loneliness.

## Conclusions

The study showed that nearly half of the participants felt lonely. Living conditions, family size, self-rated health status, chronic illness, sleep duration, impairment, and social support were significantly associated with loneliness.

## Data Availability

The original contributions presented in the study are included in the article/Supplementary Material, further inquiries can be directed to the corresponding author.
