# Supplementary figures and images for: Prevalence of loneliness and associated factors among older adults at Yilmana Densa District, West Gojjam Zone Amhara region, Ethiopia
Source: Front Epidemiol. 2025 May 21;5:1545342. doi: 10.3389/fepid.2025.1545342 (PMC12133903; doi:10.3389/fepid.2025.1545342)

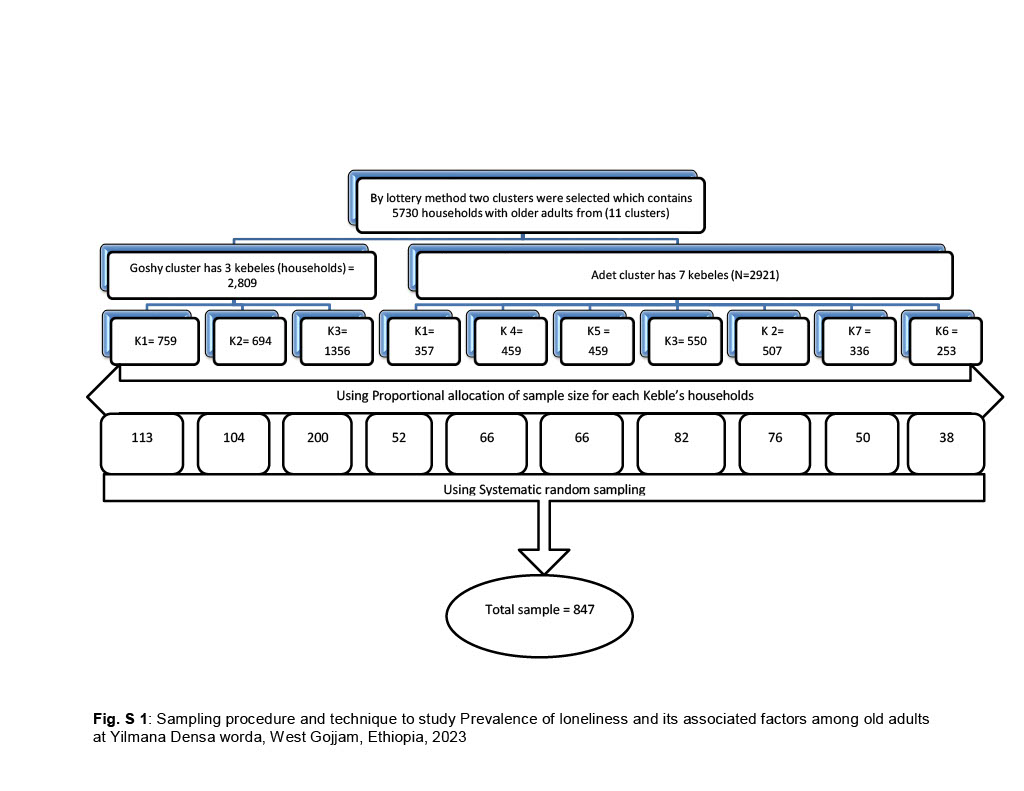

Supplement: Supplementary file 1 [file Image1.jpeg]
